# Analysis of “Dr Ding Xiang” on WeChat in China to Determine Factors Influencing Readership on Medical Social Media: Observational Study

**DOI:** 10.2196/65372

**Published:** 2025-01-20

**Authors:** Jiaman Liao, Xueliang Huang, Hao Huang, Cuina Shen, Lixia Li, Yushao Li, Yiqiang Zhan

**Affiliations:** 1 Nanlian Community Health Service Center Shenzhen Longgang Central Hospital Shenzhen China; 2 Department of Epidemiology School of Public Health Sun Yat-Sen University Shenzhen China

**Keywords:** WeChat Official Accounts, Dr Ding Xiang, health communication, information dissemination, readership analysis

## Abstract

**Background:**

With the rapid expansion of social media platforms, the demand for health information has increased substantially, leading to innovative approaches and new opportunities in health education.

**Objective:**

This study aims to analyze the characteristics of articles published on the “Dr Ding Xiang” WeChat official account (WOA), one of the most popular institutional accounts on the WeChat platform, to identify factors influencing readership engagement and to propose strategies for enhancing the effectiveness of health information dissemination.

**Methods:**

A total of 5286 articles published on the “Dr Ding Xiang” WOA from January 2021 to December 2021 were collected and analyzed. Additionally, a random sample of 324 articles was selected for detailed text analysis. Univariate analysis was conducted using the chi-square test, and multivariate analysis was performed using multivariable logistic regression.

**Results:**

In 2021, the total number of reads for “Dr Ding Xiang” articles reached 323,479,841, with an average of 61,196 reads per article. Articles exceeding 100,000 reads accounted for 33.90% of the total. Most articles were published during the time slots of 8:00-10:00 AM, 11:30 AM to 1:30 PM, and 8:30-10:30 PM. Analysis indicated that the order of publication, style of the title sentence, number of likes, number of in-views, total likes on comments, and number of replies to comments were significantly associated with an article’s number of reads. Text analysis further revealed that the article’s reasoning approaches and concluding methods also had a significant impact on readership.

**Conclusions:**

To enhance readership and the effectiveness of health communication, health-focused WOAs should consider key factors such as optimal publication timing, engaging title design, and effective content structuring. Attention to these elements can improve user engagement and support the broader dissemination of health information.

## Introduction

The rapid expansion of social media has significantly increased the demand for accessible health information, fostering innovative approaches in health education [1-3]. Facebook is widely used to disseminate health content and build community support, especially in areas like chronic disease management, vaccination campaigns, and mental health [4]. Public health organizations, such as the Centers for Disease Control and Prevention and the World Health Organization, use Facebook to establish digital platforms promoting preventive measures and healthy lifestyles [5]. Twitter’s hashtag feature enhances its capacity for real-time communication in health education and emergency responses [6]. For example, during the COVID-19 pandemic, hashtags like #COVID19 and #HealthTips facilitated quick access to information and enabled user engagement [7]. YouTube has become a leading platform for health education videos on topics such as cardiopulmonary resuscitation, vaccines, and nutrition, with organizations and health influencers producing content to improve public understanding through visual demonstrations [8].

In contrast to Western social media, WeChat has become the leading channel for health communication in China, owing to its unique privacy features, strong social networking capabilities, and multifunctionality. Since its launch by Tencent in 2011, WeChat has evolved beyond a communication tool to offer social networking, marketing, media sharing, and utilities, making it an adaptable platform for mobile health engagement [9].

The WeChat official account (WOA) feature, introduced in 2012, allows organizations, businesses, and individuals to create public accounts to broadcast information and engage users. Through WOAs, users can receive notifications, read articles, engage with participatory content, and access various services [10]. This feature has been widely adopted for health information dissemination. For instance, the Wuxi Center for Disease Control and Prevention achieved high engagement by sharing articles on nutrition and food safety, highlighting that targeted content and strategic layouts can enhance reader interaction [11]. Similarly, health programs on WeChat, such as those aimed at improving parental health literacy, underscore the platform’s effectiveness in delivering personalized health education [12].

WeChat's engagement features, including likes and comments, amplify user participation and promote the spread of health information. Studies suggest that user engagement, indicated by liking behavior, is closely linked to interest and approval [10]. WOAs are also instrumental in chronic disease management and patient education; for example, a WeChat-based management program for cough-variant asthma demonstrated significant clinical success [13]. Moreover, a life review program for patients with cancer highlighted WeChat’s potential to provide psychological support and enhance quality of life [14]. During the COVID-19 pandemic, WeChat played a crucial role in health information dissemination and public health management [15], and was used effectively to promote health behaviors, such as encouraging HIV testing [16].

Overall, WeChat has demonstrated its effectiveness as a tool for health communication and education, providing a robust platform for enhancing public health literacy, improving disease management, and delivering essential psychological support. However, there is a need for further research into optimal posting strategies and the dissemination effects of WOA articles. Addressing these gaps is crucial for understanding dissemination effectiveness and refining posting strategies. Consequently, this study conducts both a general and detailed text analysis of the articles published by “Dr Ding Xiang” WOA in 2021, aiming to explore the factors that influence readership engagement with health-related WeChat articles. The goal is to provide a scientific basis for government departments to develop effective public account strategies and enhance the overall efficacy of health communication.

## Methods

### Study Materials

The “Dr Ding Xiang” WOA used in this study was established by Ding Xiangyuan, a prominent Chinese health care company founded in 2000. Ding Xiangyuan specializes in medical information services and health content, with a commitment to providing the public with scientific and authoritative health knowledge. The “Dr Ding Xiang” WOA serves as Ding Xiangyuan's public health education brand, focusing on disseminating reliable health information and enhancing public health literacy. All content is edited and reviewed by a professional team to ensure both accuracy and authority.

According to the 2019 China WeChat 500 annual list of public accounts released by the New Rank platform, “Dr Ding Xiang” ranked eighth out of 981,144 WOAs and secured the top spot among health care–related WOAs, with an average of more than 90,250 reads per article [17]. This ranking underscores the notable influence and representativeness of the “Dr Ding Xiang” WOA in disseminating health information. Therefore, a comprehensive analysis was conducted on 5286 articles published by the “Dr Ding Xiang” WOA from January 2021 to December 2021. Additionally, a sample of 324 articles was randomly selected for in-depth text analysis, using simple random sampling to ensure representativeness.

### Data Collection

For the main analysis, data were sourced from the New Rank platform [18], accessed on September 20, 2022, a recognized authority in China for assessing the data value of WOA content. We collected and compiled data on 5286 articles published by “Dr Ding Xiang” in 2021. The dataset included various attributes such as time of publication, order of publication, style of the title sentence, presence of authors, originality of the articles, use of multimedia, number of likes, number of in-views, number of comments, total likes on comments, number of replies to comments, and total likes on replies to comments. This extensive dataset facilitated a detailed examination of the publication characteristics of each article.

For the text analysis, a sample of 324 articles was randomly selected to investigate specific text attributes, including the article’s thematic contents, opening styles, reasoning approaches, and concluding methods. The objective was to identify factors that significantly influence readership and engagement with these health-related articles. By analyzing these attributes, we aimed to uncover elements that most effectively enhance audience interaction and engagement, thereby providing valuable insights for crafting successful health communication strategies on WOAs.

### Variables Assessment

The time of publication refers to the exact date and time when an article is made available to the public. This grouping is based on our observations of user behavior, which indicate that peak browsing and interaction times typically occur between 8:00-10:00 AM, 11:30 AM to 1:30 PM, and 8:30-10:30 PM. These periods likely align with daily commuting, lunch breaks, and evening leisure activities, respectively.

The order of publication indicates an article placement within a multiarticle push, where position 1 is the headline and typically receives the highest exposure, with up to 8 positions available per push. The style of the title sentences is categorized based on the presence of exclamation marks and question marks. An article is deemed to have an author if the author’s name is signed beneath the title. Articles carrying an “original” tag are considered original. Beyond images and text, articles can also include videos and audio links; in this study, the inclusion of such elements is referred to as multimedia use.

Each article includes the “Like” and “In-view” buttons at the bottom right corner, from which the counts for likes and in-views are obtained. Here, it is important to clarify the specific function of “In-view” in this context. When a user clicks the “In-view” button, the article appears in the user’s WeChat “Discover > Take a look > In-view” section, making it visible to the user’s WeChat contacts. Unlike “Like,” which merely indicates user appreciation, the “In-view” feature serves as a form of recommendation, enhancing the article’s visibility among the user’s social network.

Articles also feature a commenting function, with the number of comments determined by the total number of messages left on each article. Each comment also has a “Like” button, and the sum of all likes on comments constitutes the total number of likes on comments. When readers leave comments, both the operator and other readers can reply, and these replies are counted as comment replies. If readers agree with the replies, they can also like them, and the total number of likes on replies is recorded.

On the WeChat platform, the recommendation mechanism promotes content based on article interaction data, such as the number of likes and comments. Articles that receive higher levels of interaction are more likely to be recommended to a broader audience. Additionally, behaviors such as liking and commenting often lead to social sharing, where users may forward articles to their social circles or share them directly with friends [19]. Given these dynamics, the number of likes and comments on the WeChat platform serves not only as a user’s response to the content but may also contribute to an increase in readership through both the recommendation system and social dissemination. Consequently, this study considers the number of likes and comments as potential factors influencing reading volume.

The article thematic contents included beauty and fitness (focusing on skincare, makeup, fitness, and weight loss), maternal and child health (covering obstetrics, gynecology, pediatrics, and early childhood education, targeting individuals planning to get pregnant, pregnant women, and parents of children aged 0-6 years), gender health (including topics on marriage, contraception, and sexually transmitted diseases), common diseases (covering prevalent health conditions), and non–health-related topics (such as product sales and recruitment).

The article opening styles included introductory style (introduced by the main subject, related topics, questions, or proverbs and sayings), case style (opening with personal or third-party cases), festive style (opening with mentions of festivals, seasons, or specific times of the year), social news (starting with a news event), and other styles (directly beginning with the main text content).

The article reasoning approaches used in the articles include specialized knowledge explanations (using professional knowledge to explain), data, guidelines, and experiment-based explanations (citing data, research guidelines, and experiments), case-based explanations (using specific cases to explain), and other forms (using various other forms of reasoning). To ensure consistency and comparability in categorization, articles were assigned to the appropriate category based on the predominant type of reasoning used. For instance, if an article primarily serves as an explanation of professional knowledge, supplemented by case studies, it would be classified as an “explanation of professional knowledge.”

The article concluding methods include summary endings (refining and summarizing the main points of the article), supplementary tips (reemphasizing important content and adding relevant information), call-to-action endings (encouraging the audience to take specific actions), advertising and promotional endings (promoting product purchases and live broadcasts), and other endings (a few articles simply conclude with the main text content).

### Quality Control

The data on the number of reads, number of in-views, number of likes, number of comments, total number of comments, number of replies to comments, total number of replies to comments, and other related metrics collected in this study were based on the statistics as of September 20, 2022. To ensure the accuracy of data entry for determining the article topic, opening style, reasoning form, and ending style, 2 coauthors were trained for this. They independently entered data for 324 randomly selected articles. In cases of disagreement, the final confirmation was made through discussion and consensus among a third author.

### Statistical Analysis

Articles were categorized into 2 groups based on reading volume: a high-reads group (100,000 and above reads) and a low-reads group (fewer than 100,000 reads). Independent variables included the time of publication, order of publication, style of the title sentence, presence of authors, originality of articles, use of multimedia, number of likes, number of in-views, number of comments, number of total likes on comments, number of replies to comments, number of total likes on replies to comments, article thematic contents, article opening styles, article reasoning approaches, and article concluding methods. A chi-square test was used for univariate analysis, with statistically significant variables included in a logistic regression model. A *P* value<.05 was considered statistically significant. All analyses were conducted using R (version 4.1; R Foundation for Statistical Computing). Model fit was assessed with the Hosmer-Lemeshow test, where a *P* value>.05 indicates a good fit (ie, no significant difference between observed and predicted values). To address multicollinearity, variance inflation factor (VIF) and tolerance values were calculated. A tolerance value of 0.1 or greater and a VIF value of less than 10 were considered indicators of no multicollinearity.

### Ethical Considerations

This study was conducted using publicly available data from the New Rank platform, which was accessed in accordance with the platform’s terms of service. All data included in this study were either anonymous or deidentified prior to analysis, ensuring that no personally identifiable information was collected or processed. As the study involved the observation of publicly available data and secondary analyses, no direct interaction with individuals occurred, and no formal ethical approval was required. To further protect privacy, the results are presented in aggregate form without identifying specific individuals or posts. These measures align with established ethical standards for research involving publicly accessible data.

## Results

### Basic Characteristics of Published Articles

According to real-time statistics on September 20, 2022, a total of 5286 articles were published on the “Dr Ding Xiang” WOA in 2021. On average, about 14 articles were posted daily. Each article garnered an average of approximately 61,196 reads, with the lowest recorded number being 3577 reads. Among these, 1792 (33.90%) articles surpassed 100,000 reads. The publication times of the articles were predominantly clustered into 3 periods: 8:00-10:00 AM, which saw 1733 (32.78%) articles; 11:30 AM to 1:30 PM, with 1892 articles (35.79%); and 8:30-10:30 PM, during which 1642 (31.06%) articles were published.

The articles accumulated a total of 273,712,120 likes, averaging approximately 518 likes per article. Additionally, they received 1,848,787 in-views, with an average of around 350 in-views per article. The total number of comments across all articles was 53,495, averaging about 10 comments per article. Collectively, likes on comments totaled 1,595,956, translating to an average of approximately 2856 interactions per article. The total number of replies to comments reached 20,722, averaging about 4 replies per article. Lastly, likes on replies to comments amounted to 5,573,912, with an average of roughly 1054 such interactions per article.

### Univariate Analysis

The univariate analysis revealed that several factors were significantly associated with the number of reads for the articles. Specifically, the order of publication (*χ*^2^_2_=4040.2; *P*<.001), style of the title sentence (*χ*^2^_3_=66.1; *P*<.001), presence of authors (*χ*^2^_1_=219.8; *P*<.001), and originality of the articles (*χ*^2^_1_=1029.6; *P*<.001) were all important determinants. Additionally, the use of multimedia (*χ*^2^_1_=31.0; *P*<.001), as well as various engagement metrics such as the number of likes (*χ*^2^_2_=2290.0; *P*<.001), in-views (*χ*^2^_2_=2506.8; *P*<.001), comments (*χ*^2^_2_=2255.3; *P*<.001), total likes on comments (*χ*^2^_2_=2397.5; *P*<.001), replies to comments (*χ*^2^_2_=1750.4; *P*<.001), and total likes on replies to comments (*χ*^2^_2_=2306.4; *P*<.001) also showed significant impacts. These findings are further detailed in Table 1.

**Table 1:** Univariate analysis of the readership of 5286 articles on “Dr Ding Xiang” WOA^a^ in 2021.

Variables			Total, n		High reads, n (%)		Low reads, n (%)		*P* value	
**The time of publication**										.25
	8:00-10:00 AM	1733		1171 (67.6)		562 (32.4)				
	11:30 AM to 1:30 PM	1892		1245 (65.8)		647 (34.2)				
	8:30-10:30 PM	1642		1068 (65)		574 (35)				
	Other time slots	19		10 (52.6)		9 (47.4)				
**The order of publication**										<.001
	In the first to second positions	1877		194 (10.3)		1683 (89.7)				
	In the third to fifth positions	2600		2503 (96.3)		97 (3.7)				
	In the sixth to eighth positions	809		797 (98.5)		12 (1.5)				
**The style of the title sentence**										<.001
	Declarative sentences	1828		1091 (59.7)		737 (35.3)				
	Interrogative sentences	2027		1461 (72.1)		566 (27.9)				
	Exclamatory sentences	1207		797 (66)		410 (34)				
	Interrogative exclamatory sentences	224		145 (64.7)		79 (35.3)				
**The presence of authors**										<.001
	Not have	875		768 (87.8)		107 (12.2)				
	There are	4411		2726 (61.8)		1685 (38.2)				
**The originality of the articles**										<.001
	Original	4006		3121 (77.9)		885 (22.1)				
	Not original	1280		373 (29.1)		907 (70.9)				
**The use of multimedia**										<.001
	Not used	5117		3416 (66.8)		1701 (33.2)				
	Used	169		78 (46.2)		91 (53.8)				
**The number of likes**										<.001
	0-100	2906		2579 (88.7)		327 (11.3)				
	101-300	1338		841 (62.9)		497 (37.1)				
	301 and greater	1042		74 (7.1)		968 (92.9)				
**The number of in-views**										<.001
	0-50	2828		2588 (91.5)		240 (8.5)				
	51-150	1364		823 (60.3)		541 (39.7)				
	151 and greater	1094		83 (7.6)		1011 (92.4)				
**The number of comments**										<.001
	0-5	2473		2303 (93.1)		170 (6.9)				
	6-15	1616		1022 (63.2)		594 (36.8)				
	16 and greater	1197		169 (14.1)		1028 (85.9)				
**The number of total likes on comments**										<.001
	0-100	2457		2261 (92)		196 (8)				
	101-1000	1565		1082 (69.1)		483 (30.9)				
	1001 and greater	1264		151 (11.9)		1113 (88.1)				
**The number of replies to comments**										<.001
	0-1	2514		2233 (88.8)		281 (11.2)				
	2-5	1507		1001 (66.4)		506 (33.6)				
	6 and greater	1265		260 (20.6)		1005 (79.4)				
**The number of** **total likes on replies to comments**										<.001
	0-10	2402		2194 (91.3)		208 (8.7)				
	11-250	1594		1129 (70.8)		465 (29.2)				
	251 and greater	1290		171 (13.3)		1119 (86.7)				

^a^WOA: WeChat official account.

### Multivariable Analysis

The multivariable analysis results indicated that several factors significantly enhanced readership. The order of publication had a considerable impact, with articles posted in the first to second positions showing an odds ratio (OR) of 745.654 (95% CI 358.061-1552.809; *P*<.001) and those in the third to fifth positions showing an OR of 2.719 (95% CI 1.391-5.315; *P*=.003). The style of the title sentence also influenced readership, particularly declarative sentences (OR 2.333, 95% CI 1.572-3.461; *P*<.001) and interrogative sentences (OR 1.928, 95% CI 1.297-2.867; *P*=.001). The number of likes significantly boosted readership, with likes in the range of 101-300 (OR 2.308, 95% CI 1.384-3.848; *P*=.001) and 301 and greater (OR 14.050, 95% CI 5.949-33.184; *P*<.001). Similarly, the number of in-views positively impacted readership, particularly for in-views in the range of 51-150 (OR 2.739, 95% CI 1.736-4.321; *P*=.001) and 151 and greater (OR 4.087, 95% CI 1.617-9.197; *P*<.001). The number of total likes on comments also boosted readership, with ranges of 101-1000 likes (OR 3.082, 95% CI 2.125-4.470; *P*<.001) and 1001 and greater (OR 9.747, 95% CI 5.515-17.226; *P*<.001). Additionally, the number of replies to comments had a positive effect, especially for comments liked 1001 times and greater (OR 9.747, 95% CI 5.515-17.226; *P*<.001) and the number of replies to comments reaching 6 and greater (OR 2.374, 95% CI 1.560-3.612; *P*<.001). These factors are summarized in Table 2.

The goodness of fit tests for the fitted binary logistic regression model was greater than the significance level of .05, which indicates the model adequately fits the data. In addition, the results also show that the VIF values of all independent variables are less than 10 and the tolerance values are greater than 0.1, indicating that the effect of multicollinearity on the model is negligible.

**Table 2:** Multivariable analysis of the readership of 5286 articles of “Dr Ding Xiang” WOA^a^ in 2021.

Factor			OR^b^ (95% CI)		*P* value	
**The order of publication**						<.001
	In the sixth to eighth positions	1 (reference)		—^c^		
	In the third to fifth positions	2.719 (1.391-5.315)		.003		
	In the first to second positions	745.654 (358.061-1552.809)		<.001		
**The style of the title sentence**						<.001
	Exclamatory sentences	1 (reference)		—		
	Declarative sentences	2.333 (1.572-3.461)		<.001		
	Interrogative sentences	1.928 (1.297-2.867)		.001		
	Interrogative exclamatory sentences	1.38 (0.676-2.817)		.38		
**The number of likes**						<.001
	0-100	1 (reference)		—		
	101-300	2.308 (1.384-3.848)		.001		
	301 and greater	14.05 (5.949-33.184)		<.001		
**The number of in-views**						<.001
	0-50	1 (reference)		—		
	51-150	2.739 (1.736-4.321)		<.001		
	151 and greater	4.087 (1.817-9.197)		.001		
**The number of total likes on comments**						<.001
	0-100	1 (reference)		—		
	101-1000	3.082 (2.125-4.470)		<.001		
	1001 and greater	9.747 (5.515-17.226)		<.001		
**The number of replies to comments**						<.001
	0-1	1 (reference)		—		
	2-5	1.208 (0.845-1.727)		.30		
	6 and greater	2.374 (1.560-3.612)		<.001		

^a^WOA: WeChat official account.

^b^OR: odds ratio.

^c^Not applicable.

### Text Analysis for the Selected Articles

The text analysis of the selected articles revealed a diverse distribution of topics. Beauty and fitness were the most common topics, comprising 80 (24.7%) articles, followed by common diseases with 77 (23.8%) articles. Maternal and child health, gender health, and topics unrelated to health accounted for 20 (6.2%) articles, 22 (6.8%) articles, and 95 (29.3%) articles, respectively. Regarding the opening form, introductions were prevalent in 203 (62.7%) articles, while case studies were used in 54 (16.7%) articles. Articles that began with festivals, social news, or other forms made up 22 (6.8%) articles, 18 (5.6%) articles, and 27 (8.3%) articles, respectively. In terms of reasoning forms, specialized knowledge was used in 160 (49.4%) articles, followed by data citation, research guidelines, and experiments in 47 (14.5%) articles. Experiments constituted the reasoning form in 12 (3.7%) articles, case studies in 40 (12.3%) articles, and other reasoning forms in 77 (23.8%) articles. As for the conclusions, 26 (8%) articles provided a summary, while hints and calls for action were each present in 42 (13%) articles. A significant number of articles concluded with advertisements and promotions, totaling 178 (54.9%) articles, with 36 (11.1%) articles using other forms of conclusions.

### Univariate and Multivariate Analyses

The univariate analysis showed that the article opening styles (*χ*^2^_4_=10.6; *P*=.03), the article reasoning approaches (*χ*^2^_3_=23.7; *P*<.001), and the article concluding methods (*χ*^2^_4_=22.4; *P*<.001) significantly affected article readership, as detailed in Table 3.

**Table 3:** Univariate analysis of the readership of 324 articles on “Dr Ding Xiang” WOAa in 2021.

Variables			Total, n		High reads, n (%)		Low reads, n (%)		*P* value	
**The article thematic contents**										.08
	Beauty and fitness	80		40 (50)		40 (50)				
	Maternal and child health	50		35 (70)		15 (30)				
	Gender health	22		11 (50)		11 (50)				
	Common disease	77		52 (67.5)		25 (32.5)				
	Non–health-related	95		54 (56.8)		41 (43.2)				
**The article opening styles**										.03
	Introductory style	203		123 (60.6)		80 (39.4)				
	Case style	54		23 (43.6)		31 (57.4)				
	Festive style	22		13 (59.1)		9 (40.9)				
	Social news	18		12 (66.7)		6 (33.3)				
	Other styles	27		21 (77.8)		6 (22.2)				
**The article reasoning** **approaches**										<.001
	Specialized knowledge explanations	160		112 (70)		48 (30)				
	Data, guidelines, and experiment-based explanations	47		21 (44.7)		26 (55.3)				
	Case-based explanations	40		13 (32.5)		27 (67.5)				
	Other forms	77		46 (59.7)		31 (40.3)				
**The article concluding methods**										<.001
	Summary endings	26		7 (26.9)		19 (73.1)				
	Supplementary tips	42		25 (59.5)		17 (40.5)				
	Call to action endings	42		19 (45.2)		23 (54.8)				
	Advertising and promotional endings	178		112 (62.9)		66 (37.1)				
	Other endings	36		29 (80.6)		7 (19.4)				

^a^WOA: WeChat official account.

In the multivariate analysis, it was evident that specific reasoning approaches and concluding methods had substantial impacts on readership engagement. Articles citing data, guidelines, and experiment-based explanations (OR 2.288, 95% CI 1.132-4.623; *P*=.02), using case-based explanations (OR 4.713, 95% CI 2.169-10.237; *P*<.001), notably boosted readership. Conversely, certain concluding methods such as supplementary tips (OR 0.267, 95% CI 0.089-0.805; *P*=.02), Advertising and promotional endings (OR 0.243, 95% CI 0.092-0.641; *P*=.004), and other endings (OR 0.094, 95% CI 0.027-0.327; *P*<.001) were found to inhibit readership. These findings are comprehensively summarized in Table 4.

The goodness of fit tests for the fitted binary logistic regression model was greater than the significance level of .05, which indicates the model adequately fits the data. In addition, the results also show that the VIF values of all independent variables are less than 10 and the tolerance values are greater than 0.1, indicating that the effect of multicollinearity on the model is negligible.

**Table 4:** Multivariate analysis of the readership of 324 articles on “Dr Ding Xiang” WOA^a^ in 2021.

Variables			OR^b^ (95% CI)		*P* value
**The article reasoning** **approaches**					
	Specialized knowledge explanations	1 (reference)		—^c^	
	Data, guidelines, and experiment-based explanations	2.288 (1.132-4.623)		.02	
	Case-based explanations	4.713 (2.169-10.237)		<.001	
	Other forms	1.828 (1.006-3.320)		.05	
**The article concluding methods**					
	Summary endings	1 (reference)		—	
	Supplementary tips	0.267 (0.089-0.805)		.02	
	Call to action ending	0.459 (0.153-1.372)		.16	
	Advertising and promotional endings	0.243 (0.092-0.641)		.004	
	Other endings	0.094 (0.027-0.327)		<.001	

^a^WOA: WeChat official account.

^b^OR: odds ratio.

^c^Not applicable.

## Discussion

### Principal Results

Our study reveals that headline and subheadline articles achieve significantly higher readership compared to subordinate articles. This is substantiated by previous research indicating that the first article typically garners the highest attention, with subsequent articles experiencing a sharp decline in readership. This can be attributed to the initial engagement creating a strong first impression, which diminishes as readers move through additional content [20]. Hence, crafting compelling headlines and engaging thumbnails becomes critical in capturing and retaining reader attention. These elements act as the primary hooks that encourage readers to click on and continue through the content, thereby enhancing the overall readership of subsequent articles [21].

Survey data from China provide insights into reader behavior, revealing that most readers skim the body text after reading the headline, highlighting the headline’s pivotal role in capturing initial interest [22]. Our analysis emphasizes the importance of linguistic choices in headlines. Studies show that using emotion-laden words, third-person pronouns, and clear definitive articles can significantly enhance click-through rates by appealing to readers’ emotions and curiosity [23]. Specifically, interrogative headlines, which pose questions, are particularly effective in engaging readers as they evoke a sense of suspense and intrigue. This finding aligns with research from ScienceDaily and Azura Magazine, which suggests that questions naturally stimulate curiosity and engagement [24,25].

However, titles with an exclamatory tone, often perceived as exaggerated or sensational, can lead readers to question the credibility of the content, thereby diminishing their trust. Research on health education communication highlights trust as a crucial factor in effective information dissemination [26]. While dramatic language and punctuation may capture attention quickly, they can also give the impression that the information is unreliable or inaccurate.

To address this, declarative or interrogative titles are recommended for conveying the article’s core content authentically and effectively. Declarative titles clearly communicate the main idea, set accurate expectations, and establish a direct communication style, which is critical for building trust in health-related content. Conversely, interrogative titles stimulate curiosity by posing questions, encouraging readers to explore the content further without compromising its credibility. By fostering trust through accurate and engaging titles, authors can enhance reader satisfaction and promote meaningful, long-term interactions with health information.

The study highlights a significant relationship between article reads, likes, and engagement in the comment sections. This relationship may not only reflect users’ interest in the content but may also indirectly impact reading volume through WeChat’s recommendation mechanism and users' social sharing behavior. Driven by this recommendation mechanism, articles with higher interaction data receive greater exposure, while users’ liking and commenting behavior can further stimulate retweeting within their social circles, thereby expanding the reach of the articles. Consequently, on the WeChat platform, engagement behaviors may serve both as a reflection of reading and as a potential influencing factor in enhancing reading volume.

This study’s classification system is based on the varying impacts of reasoning types in articles published on WOAs [27]. Our findings indicate that explanations based on data, guidelines, experiments, cases, or other forms were read more frequently than specialized knowledge explanations. These formats make the content more relatable and easier to understand. Furthermore, summary endings were preferred over supplementary tips and advertising and promotional endings, as they save readers' time, emphasize key points, and enhance the content's value, retention, and shareability [28,29].

The insights derived from this study hold critical implications for public health promotion and information dissemination. Effectively engaging readers through strategic headline crafting, linguistic choices, and participatory features can significantly enhance the reach and impact of health messages. By adopting these strategies, public health communicators can ensure that vital health information is disseminated widely and engaged with meaningfully, thereby promoting healthier behaviors and outcomes within the community.

### Limitations

The strengths of this study lie in its empirical basis, comprehensive data collection over a 1-year period, and the bifurcated approach of general and text analysis, providing a well-rounded investigation of factors influencing readership. However, the study also has limitations. It focuses on a single public account, restricting the analysis to a longitudinal perspective without cross-sectional validation against other health-related WOAs. Additionally, WeChat readership metrics cap at 100,000 reads, preventing accurate assessment for articles exceeding this threshold and thus limiting readership as a true continuous variable. To address this, we dichotomized readership into “high reads” (100,000 reads and more) and “low reads” (less than 100,000 reads) for analysis. Finally, the text analysis relies on subjective definitions of the article’s thematic contents, the opening styles, the reasoning approaches, and the concluding methods, which may introduce bias despite the random article selection.

### Conclusions

In summary, the study offers valuable insights into effective strategies for enhancing readership and engagement, which are crucial for successful health communication and promotion. By understanding and leveraging these factors, public health professionals can better capture and retain reader interest, ensuring that essential health information reaches and resonates with a broader audience.

## Abbreviations

OR: odds ratio
VIF: variance inflation factor
WOA: WeChat official account

## Data Availability

The datasets generated and/or analyzed during this study are not publicly available due to privacy concerns and ethical considerations related to user interaction metrics (eg, likes, comments, and views) but are available from the corresponding author on reasonable request.
